# ABT Promotes Adventitious Root Formation in Mulberry Cuttings by Coordinating Hormonal Homeostasis and Defense Priming

**DOI:** 10.3390/cimb48030299

**Published:** 2026-03-11

**Authors:** Zhen Qin, Tiantian Wang, Ziyi Song, Hao Dou, Chaobing Luo, Xiu Zhang, Huijuan Sun, Bingyang Zhang, Yaru Hou, Shihao Sun, Chenbo Tan, Jin’e Quan, Zhaojun Liu

**Affiliations:** 1 College of Forest, Henan Agricultural University, Zhengzhou 450002, China; 15670228779@163.com (Z.Q.); wangtian@stu.henau.edu.cn (T.W.);; 2 Biological Breeding Laboratory, Xinjiang Uygur Autonomous Region ‘Academy’ of Agricultural Sciences, Urumgi 830091, China

**Keywords:** ABT, mulberry, cutting propagation, adventitious root, hormonal homeostasis, defense response, transcriptome

## Abstract

Mulberry (*Morus alba*) is an economically important forest tree species, yet cutting propagation is constrained by low adventitious rooting efficiency. Although ABT, a composite rooting promoter, can improve cutting survival, its molecular basis remains unclear. Here, cuttings of the cultivar Qiangsang 1 were treated with ABT, NAA, or IAA (200–1000 mg/L) and subjected to transcriptome profiling to elucidate how ABT enhances rooting. Hormone-related analyses showed that ABT upregulated GH3 (auxin-amido synthetase) at days 0 and 20, implicating auxin homeostasis. ERF1/2 (ethylene response factors) exhibited a temporal oscillation, with induction at day 10 followed by repression from days 20 to 30, consistent with a shift from developmental programs to defense-related processes. In parallel, JAZ (jasmonate ZIM-domain) genes were downregulated at day 0 and subsequently upregulated; together with CYP94C1, these changes may attenuate jasmonate-associated defense signaling. For cell remodeling and defense coordination, ABT reduced the expression of genes associated with cell-wall rigidity while inducing EXPA11 (expansin) at day 20, potentially facilitating root primordium emergence. Meanwhile, PR-1 (pathogenesis-related protein 1) was transiently upregulated at days 0, 20, and 30, and the concomitant modulation of WRKY transcription factors and RPM1 suggests enhanced defense readiness. Integrative network analysis further indicated that a GH3–ERF1/2–PR-1 module links hormonal and defense cues and may activate BAT1 (energy metabolism) and RBOHB (ROS production) to support adventitious root elongation. Collectively, these results suggest that ABT improves rooting efficiency by reshaping hormonal homeostasis and coordinating cell-wall reconstruction with a pre-activated defense state, thereby providing a conceptual framework for balancing root induction and defense responses during vegetative propagation in forest trees.

## 1. Introduction

Cutting is a key method for vegetative propagation in woody plants [1]. However, the low efficiency of adventitious root (AR) formation remains a major bottleneck for many woody species [2]. Therefore, improving AR formation in cuttings is a critical goal for the horticulture and forestry industries, which depend on the large-scale production of reliably rooting propagules. AR formation is an organogenic process in which competent or partially differentiated cells from stems, leaves, or hypocotyls initiate new roots, and it can be triggered by endogenous developmental cues as well as external environmental stimuli [3]. For example, mechanical injury, waterlogging, low light intensity, and changes in soil nutrient availability can modify endogenous hormone levels and other regulatory signals, thereby promoting root initiation [4,5,6].

Previous studies indicate that multiple phytohormones regulate AR formation [7]. Among endogenous auxins, indole-3-acetic acid (IAA) is the primary natural auxin and effectively promotes AR development [8]. During the early phase of AR induction, elevated IAA levels are commonly associated with root primordium differentiation [9]. IAA typically accumulates at the cutting base during induction but decreases after root primordia are established. Ethylene also plays an important role in AR formation; moderate ethylene levels can stimulate root growth in chrysanthemum, petunia, and Arabidopsis [10,11,12]. Moreover, ethylene interacts with IAA to modulate AR formation in a species- and context-dependent manner [12,13]. Consistently, members of the ethylene-responsive AP2/ERF transcription factor family are upregulated during the AR induction stage in poplar [14]. In contrast, cytokinins often antagonize auxin signaling and inhibit AR development across diverse plant species [15,16,17]. For example, in apple (Malus domestica Borkh.), exogenous application of the cytokinin analog 6-benzyladenine suppresses root primordium formation in the M.26 rootstock. Gibberellins generally act as negative regulators of AR formation [18]; in rice, exogenous gibberellin reduces AR number, whereas gibberellin-biosynthesis mutants produce more ARs [19]. In addition, gibberellins can suppress AR formation in Arabidopsis and poplar by disrupting polar auxin transport [20].

Plant growth regulators are widely applied to enhance cutting propagation [21]. Rooting formulations such as ABT (20% NAA and 30% IAA) have been reported to substantially improve rooting performance under specific conditions. In mulberry, treatment with 0.10–0.13% indole-3-butyric acid (IBA) or ABT rooting powder achieved survival rates exceeding 81%. In addition, several studies suggest that combined applications of naphthaleneacetic acid (NAA) and IAA can outperform NAA alone for certain rooting-related traits. Consistently, comparisons of IAA and ABT—applied individually or in combination—showed that the combined treatment conferred greater benefits than either regulator alone [22,23,24,25].

Mulberry is often regarded as a difficult-to-root species, yet ABT treatment markedly enhances adventitious rooting. However, the molecular mechanisms by which ABT promotes adventitious root (AR) formation in mulberry cuttings remain poorly understood. Here, we analyzed transcriptomes from cuttings treated with 1000 mg/L ABT and applied weighted gene coexpression network analysis (WGCNA), Gene Ontology (GO) enrichment, and Kyoto Encyclopedia of Genes and Genomes (KEGG) pathway analysis to characterize ABT-associated transcriptional changes at the cutting base. These analyses focused on hormone signaling, cell-wall remodeling, and defense-related responses. Our findings provide mechanistic insight into the action of composite plant growth regulators and identify candidate genes that may support the development of more efficient vegetative propagation technologies for forest trees.

## 2. Materials and Methods

### 2.1. Experimental Site, Plant Materials, and Experimental Design

This experiment was conducted in a greenhouse at the Third Living Area of Henan Agricultural University (Zhengzhou, Henan Province, China; 113.22° E, 34.28° N; 98 m a.s.l.). The greenhouse was equipped with full-spectrum lighting and an automated misting system, and environmental conditions were maintained at 20–29 °C with 80–90% relative humidity. The cutting facility was divided into five zones (approximately 11 m × 6 m each). Each zone contained sub-beds measuring 6 m × 2 m with a depth of 0.4 m. The cutting bed was 20 m long and 1.5 m wide, and a 30 cm layer of clean river sand was used as the rooting substrate. Prior to cutting insertion, the substrate was disinfected by evenly spraying an 800-fold dilution of 50% carbendazim wettable powder until the sand was fully moistened, followed by air-drying for 3 days. On day 4, the prepared cuttings were briefly immersed (10 s) in the same carbendazim dilution before planting. Mulberry cuttings were provided by the research group of Quan Jin’e at Henan Agricultural University.

**Figure 1:**
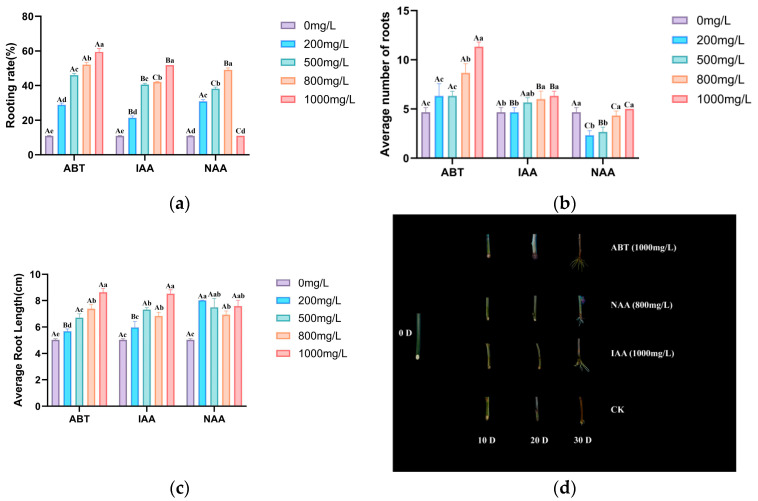
Effects of Different Growth Regulator Concentrations on Mulberry Cuttings. (**a**) Rooting percentage (%), (**b**) root number (roots), and (**c**) root length (cm) under treatments with ABT, IAA, or NAA at 0–1000 mg/L. Data are means (n = 3). Two-way ANOVA followed by Duncan’s multiple range test was performed. Note: Uppercase letters compare hormone types at the same concentration; lowercase letters compare concentrations within the same hormone. Different letters indicate significant differences at *p* < 0.05. (**d**) In mulberry cuttings treated with ABT, NAA, and IAA at concentrations of 200–1000 mg/L, along with phenotypic changes observed at the cutting site over 0–30 days.

Semi-hardwood shoots of the mulberry cultivar ‘Qiangsang 1’ (*Morus* spp.), developed by the Sericulture Research Institute of the Zhejiang Academy of Agricultural Sciences, were used for the cutting trial. Shoots were collected from vigorous three-year-old plants, and current-year semi-hardwood shoots were selected for cutting preparation. Cuttings were standardized to 0.6–0.8 cm in diameter and 13–15 cm in length, with a flat apical cut and a 45° basal bevel cut. For treatments, the basal ends were immersed for 30 s in ABT, IAA, or NAA solutions at 200, 500, 800, or 1000 mg/L; control cuttings (CK) were immersed in distilled water. Each treatment included three biological replicates, with 80 cuttings per replicate (240 cuttings per treatment), all derived from the same clone. Before insertion, shoots were disinfected by soaking in carbendazim solution for 1–2 min and then air-dried in a cool, ventilated area to remove surface moisture. Treated cuttings were inserted into sterilized substrate following a previously described protocol. Rooting stages were recorded (Figure 1d) as follows: callus formation (0–10 days post-insertion), root primordium induction (10–20 days post-insertion), and adventitious root (AR) emergence (20–30 days post-insertion). To investigate AR development, cortical tissues were sampled from the basal region (~1 cm above the cut surface) of CK and ABT-treated cuttings (1000 mg/L ABT) at 0 (CK-1, ABT-1), 10 (CK-2, ABT-2), 20 (CK-3, ABT-3), and 30 days (CK-4, ABT-4) post-insertion. Samples were immediately flash-frozen in liquid nitrogen and stored at −80 °C. Twenty samples per treatment at each time point were randomly selected for transcriptome analysis.

On day 30, root number, root length, and other data were recorded for each group. GraphPad Prism 9.5 software (GraphPad Software, San Diego, CA, USA) was used for both statistical analysis and data visualization. Two-way analysis of variance (ANOVA) was performed to evaluate the main effects of hormone type and concentration, as well as their interaction, on adventitious root formation parameters (rooting rate, root number, and root length). Following ANOVA, Duncan’s multiple range test was used for post-hoc multiple comparisons to identify significant differences among treatment groups. All data are presented as mean ± standard error of the mean (SEM) from three independent biological replicates.

### 2.2. RNA Sequencing

For transcriptome sequencing (RNA-seq), three biological replicates were collected for both the control and ABT-treated groups at each time point. In total, 24 RNA-seq libraries were constructed (2 treatments × 4 time points × 3 biological replicates). Total RNA was extracted using TRIzol reagent, and library preparation and sequencing were performed by BMK (Beijing, China) on an Illumina HiSeq 2500 platform using 150 bp paired-end reads. Raw reads were filtered and aligned according to the procedure described by Ahmad.

### 2.3. Sequence Alignment to the Morus Notabilis Genome and RNA-Sequencing Data Analysis

After sequencing, raw reads were filtered by removing adapter sequences, low-quality reads, and reads containing ambiguous bases (N), resulting in high-quality clean reads. During this process, quality metrics including Q20 and Q30 scores, GC content, and sequence duplication levels were also assessed. The clean reads were then aligned to the mulberry (Morus notabilis) reference genome (https://morus.biodb.org/browse, accessed on 18 December 2024) using HISAT2. Differentially expressed genes (DEGs) were identified based on log_2_ fold changes (FC) in gene expression between different treatment stages. Genes with |log_2_(FC)| ≥ 1 and *p* ≤ 0.05 were considered as putative DEGs. DEG detection was further performed using the DESeq package in R, with the threshold set at |log_2_Ratio| ≥ 1 and a false discovery rate (FDR)-adjusted *p*-value ≤ 0.05. Functional enrichment analysis of transcripts and DEGs was carried out using the KEGG (www.kegg.jp, accessed on 18 December 2024) and GO (geneontology.org, accessed on 18 December 2024) databases. KEGG pathway analysis was applied to infer protein interaction networks and their potential biological roles, whereas GO enrichment analysis classified DEGs into three categories: Molecular Function, Cellular Component, and Biological Process. Significantly enriched pathways and GO terms were identified by hypergeometric tests against the genomic background. The resulting *p*-values were adjusted using FDR correction, and terms with FDR ≤ 0.05 were considered significantly enriched. Hypergeometric tests were implemented in the DESeq R package. The gene expression matrix and its annotation are provided in Table S1.

### 2.4. Weighted Gene Coexpression Network Analysis

Weighted gene coexpression network analysis (WGCNA) was conducted to explore expression correlation patterns among genes, using log_2_(FPKM + 1) values as input. The soft-thresholding power was determined based on the scale-free topology criterion, and the lowest power at which scale independence reached a plateau (or exceeded 0.8) was selected for subsequent analysis. Gene connectivity was also evaluated under different power values. Genes were clustered into modules using the dynamic tree-cutting method. A hierarchical clustering dendrogram was constructed from gene expression correlations, and gene modules were defined accordingly. Modules with highly similar eigengene expression profiles (merging threshold = 0.8) were subsequently combined, with each final module containing at least 50 genes. Significant modules were identified by eigengene analysis, and those of interest were selected for further investigation.

### 2.5. Validation of DEGs by RT–qPCR

RT-qPCR was performed to validate the transcriptome data by quantifying the expression levels of four selected key genes. The actin gene was used as the internal reference, with the following primer sequences: 5′-F: CTACCACTGCTGAACGGGAA-3′ and R: 5′-ACCTGTCCATCTGGCAACTC-3′. Gene-specific primers were designed according to the target gene sequences (Table S2), and assays were conducted using a Bio-Rad real-time PCR system. Each RT-qPCR reaction contained 10 µL of 2 × RealStar Green Fast Mixture (Genstar, Beijing, China), 1 µL of cDNA template, 0.5 µL each of forward and reverse primers (10 µmol/L), and 8 µL of ddH_2_O. The thermal cycling conditions were as follows: initial denaturation at 94 °C for 2 min, followed by 40 cycles of 94 °C for 15 s and 60 °C for 30 s (annealing/extension). Relative expression levels were calculated using the 2^−^ΔΔCt method.

The coexpression network constructed via WGCNA further integrated hormonal signaling, defense responses, and energy metabolism into a working system. The temporal transition from the ME8/ME9 modules, enriched in hormonal and defense pathways early (0 days), to the ME3 module, dominant in energy metabolism later (20 days), indicates a clear stage-specific prioritization in ABT regulation. The coordinated upregulation of key genes *BAT1* (energy metabolism) and *RBOHB* (ROS production) during root primordium emergence is functionally significant: *BAT1* provides energy for cell division and elongation, while *RBOHB*-generated ROS may act as secondary messengers to further amplify defense signals and potentially participate in cell-wall loosening. This efficient integration of the “energy-defense” network, from a systems biology perspective, may underlie the observed advantages of ABT over single auxins under the conditions tested—it suggests coordination between developmental, defense-related, and metabolic transcriptional responses under resource-limited conditions.

## 3. Results

### 3.1. Effects of Different Hormones on Adventitious Root Growth During Mulberry Cuttings

Previous studies have shown that exogenous auxin application can significantly enhance adventitious root (AR) formation during cutting propagation in a concentration-dependent manner. Consistent with these findings, our results demonstrated that treatments with ABT, NAA, or IAA at varying concentrations (200–1000 mg/L) significantly increased the rooting rate, mean root number, and mean root length in mulberry cuttings, compared with the control (CK, 0 mg/L) (Figure 1). Two-way ANOVA revealed significant main effects of hormone type, concentration, and their interaction on all three rooting parameters (*p* < 0.05). Data are presented as means from three independent biological replicates.

Within the ABT treatments, 1000 mg/L produced the highest rooting rate (59.42%), the greatest mean root number (11.33), and the longest mean root length (8.63 cm). For NAA, the maximum rooting rate (49.50%) occurred at 800 mg/L, whereas the highest mean root number (5.00) and mean root length (8.02 cm) were observed at 1000 mg/L and 200 mg/L, respectively. For IAA, 1000 mg/L simultaneously maximized the rooting rate (51.78%), mean root number (6.33), and mean root length (8.51 cm). Based on these results, the optimal concentrations were identified as 1000 mg/L for ABT, 800 mg/L for NAA, and 1000 mg/L for IAA, with ABT exhibiting a significantly stronger promotive effect on AR formation than NAA or IAA (Figure 1).

### 3.2. High-Throughput Gene Expression Profiling Analysis

**Figure 2:**
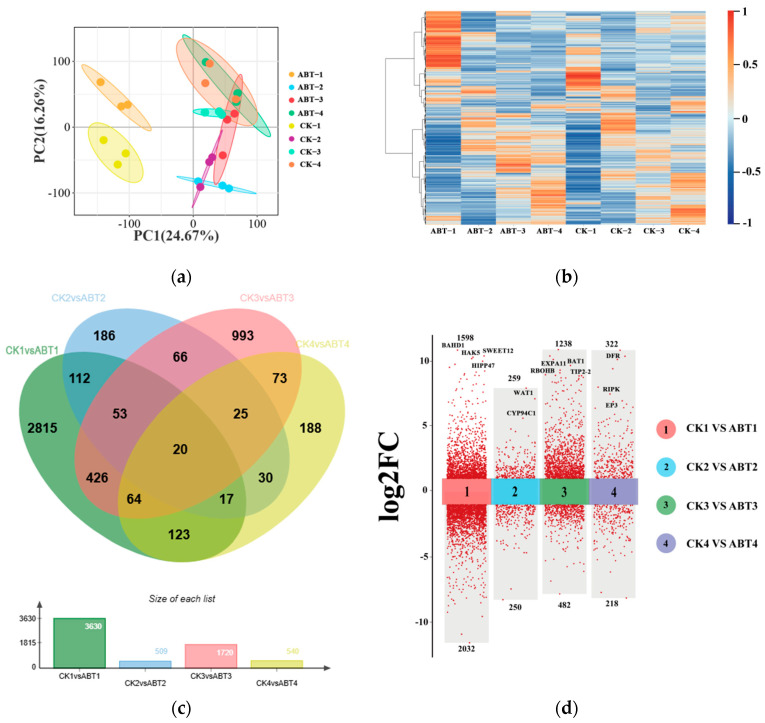
Transcriptome profiling of gene expression in mulberry cuttings in response to ABT treatment: (**a**) Principal component analysis (PCA) of gene expression data for the treatment and control groups at four time points. (**b**) Clustered heatmap of gene expression data for the treatment and control groups across four time points. (**c**) Venn diagram based on DEGs in the treatment and control groups across four time points. (**d**) The number of DEGs in the control versus treatment groups at each time point is displayed as a grouped scatter plot.

To elucidate the molecular basis by which ABT promotes adventitious root formation in mulberry, we performed RNA-seq to identify differentially expressed genes (DEGs). The experiment included four ABT-treated groups (ABT-1/2/3/4 sampled at 0, 10, 20, and 30 days, respectively) and four corresponding controls (CK-1/2/3/4 at the same time points). RNA-seq of 24 libraries generated 161.0013 Gb of high-quality clean data, with Q30 ≥ 92.26%. Principal component analysis (PCA) showed a clear separation between ABT-1 and CK-1 at day 0 (Figure 2a), indicating that ABT induces substantial transcriptomic changes at the early stage. Hierarchical clustering of genes with FPKM > 2 (FPKM, fragments per kilobase of exon model per million mapped fragments) further distinguished ABT-treated samples from controls (Figure 2b). Using count-based DEG analysis with thresholds of ∣log_2_FC∣ > 1 and FDR-adjusted *p* < 0.05, we identified 3630, 509, 1720, and 540 DEGs at 0, 10, 20, and 30 days, respectively (Table S3). Venn analysis revealed that many DEGs were shared across multiple time points (Figure 2c).

A multi-component volcano plot (Figure 2d) summarized differential expression across time points. At day 0, 1598 genes were upregulated, and 2023 were downregulated; representative genes with strong differential signals included *BAHD1* (gene15107), *HAK5* (gene3807), *SWEET12* (gene6598), and *HIPP47* (gene4437). At day 10, 259 genes were upregulated, and 250 were downregulated. Notably, *WAT1* (gene18381), which has been implicated in auxin homeostasis and secondary cell-wall formation [26], and *CYP94C1* (gene2914), which contributes to jasmonate signal attenuation via JA-Ile hydroxylation [27,28], showed pronounced differential expression. At day 20, 1238 genes were upregulated, and 482 were downregulated; prominent candidates included *EXPA11* (gene12053), reported to promote root elongation through cell-wall loosening [29], *BAT1* (gene16992), associated with energy metabolism [30], and *RBOHB* (gene17106), an NADPH oxidase involved in reactive oxygen species (ROS) production and linked to defense and developmental regulation [31,32]. At day 30, 322 genes were upregulated, and 218 were downregulated, including *RIPK* (gene10519), a central regulator of stress signaling [33], and *EP3* (gene6745), implicated in antifungal defense and potentially in developmental regulation [34]. In addition, RT-qPCR validation of four randomly selected genes showed strong agreement with the RNA-seq results (Figure S1, online Supplementary Materials).

### 3.3. Weighted Gene Coexpression Network Analysis

**Figure 3:**
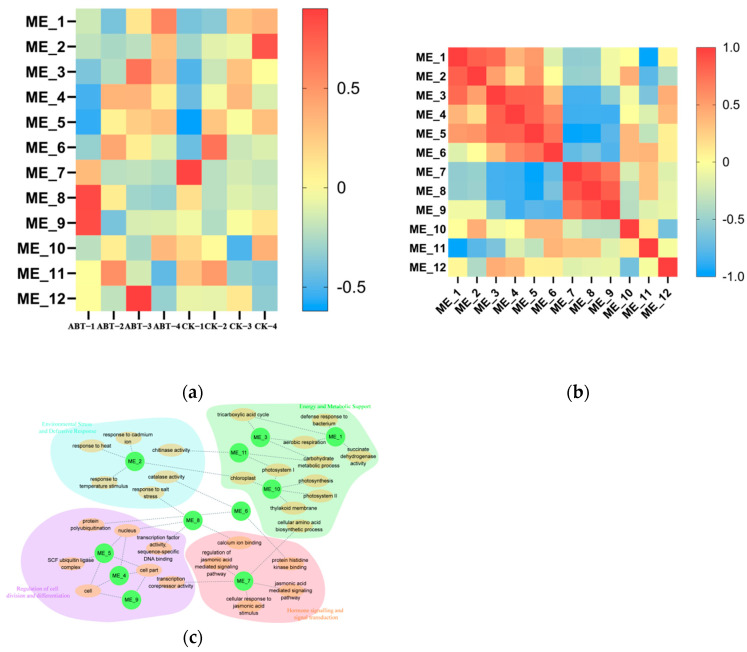
Gene Ontology (GO) analysis was performed on the transcriptome module obtained from weighted gene coexpression network analysis (WGCNA): (**a**) Analysis of the correlation between treatments and modules. The x-axis represents the treatments at different time points, and the y-axis corresponds to the distinct modules identified by WGCNA. (**b**) Correlation analysis between modules. (**c**) Integrated network of the transcript modules. The green ovals represent the WGCNA transcript modules (ME). Orange circles connected by dashed lines to MEs represent the nodes from the GO terms enriched in each module (false discovery rate < 0.05).

To elucidate the gene network underlying ABT-regulated rooting in mulberry cuttings, we performed weighted gene coexpression network analysis (WGCNA) and identified 12 coexpression modules (ME1–ME12) based on expression profiles (Figure 3a; Table S4). Module–treatment association analysis showed that ME8 and ME9 were most strongly correlated with ABT-1 (day 0), ME12 with ABT-3 (day 20), ME7 with CK-1 (day 0), and ME2 with CK-4 (day 30). Inter-module correlation analysis (Figure 3b) revealed significant positive correlations among ME3, ME4, and ME5, and among ME7, ME8, and ME9, whereas ME1 and ME11 were negatively correlated. Gene Ontology (GO) enrichment analysis (Q < 0.05) classified module genes into Molecular Function, Biological Process, and Cellular Component categories, and the resulting functional network (Figure 3c) grouped enriched terms into four domains: regulation of cell division and differentiation, hormone signal transduction, energy and metabolic support, and environmental stress/defense responses. Notably, ME8 and ME9 were upregulated in ABT-1 relative to CK-1 and were enriched for terms related to cell division/differentiation, hormone signaling, and stress/defense responses. In contrast, ME3 was primarily associated with energy and metabolic processes and showed its strongest expression differences at day 20, coinciding with adventitious root emergence and elongation, suggesting a potential contribution of metabolic support to ABT-induced rooting.

### 3.4. Effects of ABT on Hormones Within Plants

**Figure 4:**
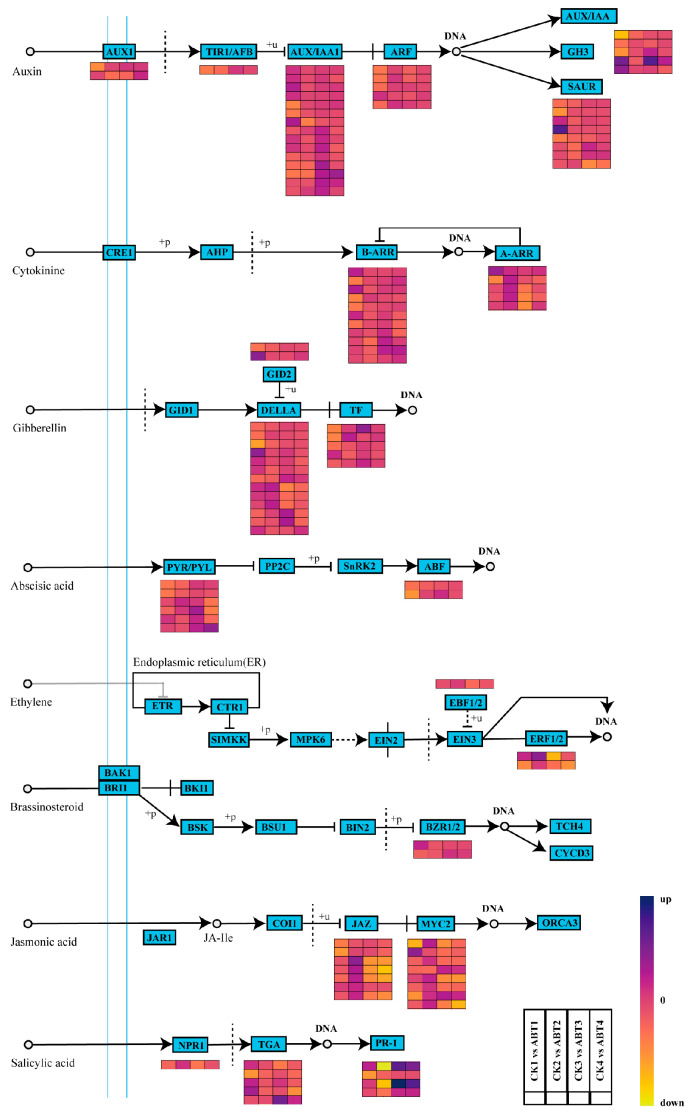
ABT affects the expression of genes related to the plant hormone signaling pathway in mulberry cuttings. The list of genes is shown in Table S5 (see online Supplementary Materials). This figure represents transcriptomic data showing gene expression changes related to the auxin signaling pathway. Pathway data were sourced from the KEGG database. Blue (33, 38, 117) indicates upregulated genes, while yellow (237, 215, 50) indicates downregulated genes.

Plant hormones are key regulators of cutting propagation. Low concentrations of gibberellin (GA), zeatin (ZT), and auxin (IAA) can promote adventitious root formation and overall plant development. Moreover, increases in the IAA/ABA or IAA/ZT ratio have been associated with accelerated callus formation and root primordium development, suggesting that adventitious root initiation is closely linked to shifts in endogenous hormonal balance. However, the hormonal mechanisms by which ABT enhances adventitious root formation remain unclear. To address this gap, we reconstructed hormone-related signaling maps based on differentially expressed genes (DEGs) and KEGG enrichment results (Figure 4).

In the auxin signaling pathway, the auxin influx carrier gene *AUX1* was upregulated in periods 3–4. *TIR1* was downregulated in periods 1–2 but became upregulated in periods 3–4. Most *AUX/IAA* genes showed sustained upregulation across the time course. *ARF* was downregulated in period 1 and upregulated in period 2. In addition, one *GH3* gene was significantly induced in periods 1 and 3, whereas one *SAUR* gene was significantly induced in period 1.

In the cytokinin signaling pathway, three *B-ARR* genes were upregulated in period 1, and *B-ARR* transcripts were broadly induced (to varying extents) in period 3. In addition, one *A-ARR* gene was significantly induced in period 1, and *A-ARR* genes were upregulated in period 2.

In the gibberellin (GA) signaling pathway, one *GID2* gene was significantly upregulated in period 1 and remained induced across subsequent periods. Among *DELLA* genes, one was significantly downregulated in period 1, three were upregulated in periods 1–2, and three others were significantly induced in period 3. In addition, three transcription factor (TF) genes were significantly upregulated, with one induced in each of periods 1, 2, and 3.

In the abscisic acid (ABA) signaling pathway, all *PYR/PYL* genes were upregulated in period 3. In addition, two *ABF* genes were downregulated in period 1 and then gradually increased in later periods.

In the ethylene signaling pathway, *EBF1/2* genes were upregulated in period 2 but downregulated in periods 3–4. The two *ERF1/2* genes showed opposite regulation in period 1 (one downregulated and the other upregulated) and displayed an increase-then-decrease pattern across subsequent periods.

In the brassinosteroid (BR) signaling pathway, one gene was upregulated in period 1, whereas two genes were upregulated in periods 3–4.

In the jasmonic acid (JA) signaling pathway, most *JAZ* genes were downregulated in period 1, upregulated in period 2, and then declined thereafter. Several *MYC2* genes showed a similar pattern, being downregulated in period 1 and upregulated in period 2; however, one *MYC2* gene was significantly induced in period 1.

In the salicylic acid (SA) signaling pathway, *NPR1* was upregulated in period 2 but downregulated at other time points. Three *TGA* genes were significantly induced in period 1, and one *TGA* gene was significantly induced in period 3. The three *PR-1* genes were significantly downregulated in period 2 but were significantly upregulated in periods 1, 3, and 4.

### 3.5. Effects of ABT on Plant–Pathogen Interactions

**Figure 5:**
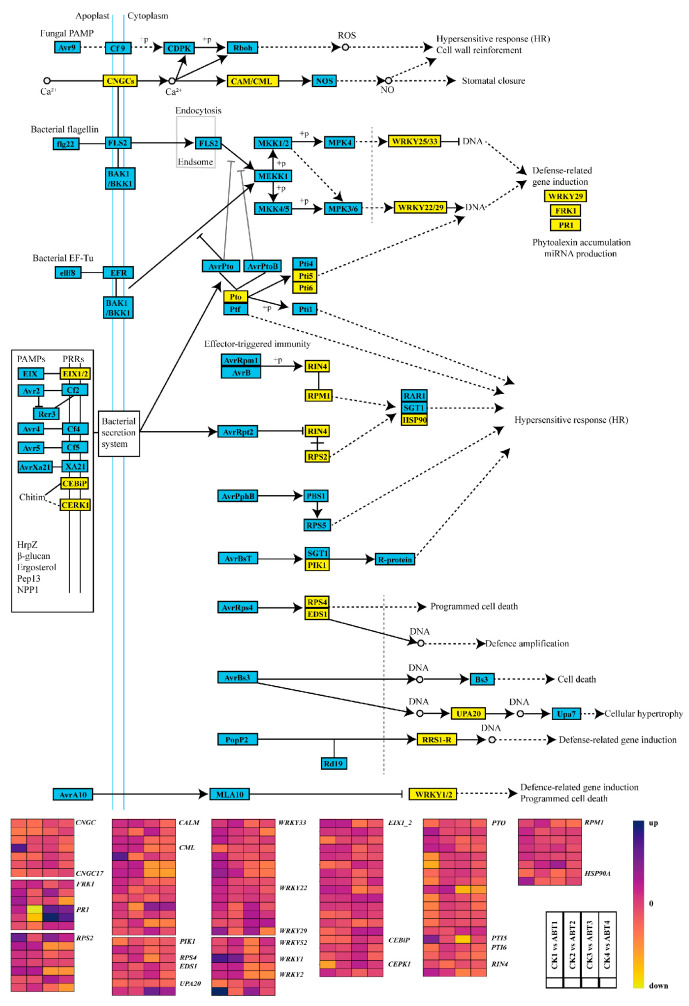
Transcriptional regulation of plant–pathogen interaction-related genes by ABT in mulberry cuttings. The list of genes is shown in Table S6 (see online Supplementary Materials). This figure represents transcriptomic data showing gene expression changes related to the plant–pathogen interaction pathway. Pathway data were sourced from the KEGG database. Blue (33, 38, 117) indicates upregulated genes, while yellow (237, 215, 50) indicates downregulated genes.

During cutting propagation, the wound surface is directly exposed to the external environment; therefore, the transcriptional defense state of cuttings may influence rooting success. However, how ABT treatment reshapes the expression of genes involved in plant–pathogen interaction pathways remains unclear. To investigate this, we performed KEGG enrichment analysis of differentially expressed genes (DEGs) and reconstructed pathogen-related pathway maps (Figure 5). Overall, defense-associated genes displayed strong temporal dynamics across the four phases.

Most *CNGC* genes were upregulated in phases 2–3, whereas only one member was significantly induced in phase 1. *CAM/CML* genes were broadly induced, with all members upregulated in phase 1, nine in phase 2, four significantly induced in phase 3, and one induced in phase 4. *WRKY25/33* genes were predominantly upregulated in phases 1–2 but were suppressed or returned to baseline levels in phases 3–4. *WRKY22/29* and *FPK1* generally showed induction in phases 1 and 3. All three *PR1* genes were upregulated in phases 1, 3, and 4 but downregulated in phase 2. Most *EIX1/2* genes showed varying degrees of upregulation across all phases, whereas all three *CEBiP* genes were induced in phase 3. *PTO* genes were upregulated in phase 2, and *PTI5* was significantly induced in phase 1 but significantly repressed in phase 3; both *PTI6* genes were induced in phase 2. All *RPM1* genes were upregulated during phases 1–3, and one *HSP90A* gene was induced in phase 1. *RPS2* genes exhibited heterogeneous patterns: four were upregulated in phases 1–2 and downregulated in phases 3–4, two were consistently upregulated across all phases, and one was downregulated in phases 1–2 but upregulated in phases 3–4. Within *UPA20*, one gene was consistently upregulated, and another was consistently downregulated across all phases. Both *RRS1-R* genes were upregulated in phases 1, 2, and 4 but downregulated in phase 3. For *WRKY1/2*, three genes were induced in phases 1–2, and one gene was induced in phases 1 and 3.

## 4. Discussion

AR formation during mulberry cutting propagation is a complex process governed by endogenous hormonal cues and modulated by environmental stress signals. Although exogenous auxin application is widely used to improve rooting, the mechanistic basis for the superior performance of composite formulations (e.g., ABT rooting powder) relative to single auxins (e.g., IAA or NAA) remains poorly defined. Guided by our hypothesis that ABT enhances rooting by coordinating hormonal signaling, cell-wall remodeling, and defense responses, we integrated phenotypic measurements with time-resolved transcriptome profiling to delineate the molecular network associated with efficient rooting. These results provide a conceptual framework for understanding how composite plant growth regulators promote vegetative propagation.

Our results indicate that ABT treatment is accompanied by pronounced transcriptional reprogramming of hormone-related signaling pathways. Consistent with reports in poplar and Arabidopsis, maintenance of auxin homeostasis is widely considered critical for successful adventitious root (AR) initiation. We detected significant upregulation of *GH3* during both the wounding stage (day 0) and the root primordium emergence stage (day 20) [35]. This pattern is consistent with the established role of *GH3* in conjugating free IAA to amino acids, thereby limiting excessive auxin accumulation and reducing potential auxin toxicity, which may help create a hormonal milieu conducive to root primordium differentiation [36].

Notably, *ERF1/2* exhibited a temporal oscillation, with an early increase followed by subsequent repression. This observation provides transcriptional support for a stage-dependent role of ethylene signaling in AR formation: moderate early activation may facilitate wound responses and cellular reprogramming, whereas later downregulation may relieve inhibitory constraints and favor root elongation [37]. Such dynamics suggest that *ERF1/2* could act as regulatory nodes integrating developmental programs with defense-associated transcriptional responses.

In addition, early downregulation of *JAZ* genes (repressors of jasmonate signaling), together with upregulation of *CYP94C1* during root primordium induction (day 10), suggests that ABT modulates both the activation and subsequent attenuation of jasmonate-related signaling. This provides a potential framework for interpreting the context-dependent roles of jasmonate in easy- versus difficult-to-root species: ABT may transiently enhance jasmonate signaling to initiate wound responses, followed by *CYP94C1*-mediated JA-Ile turnover to prevent sustained signaling that could otherwise impede rooting.

At the cellular-structural level, our results extend current understanding of cell-wall remodeling during adventitious root (AR) formation. The strong induction of *EXPA11* during root primordium emergence (day 20), together with downregulation of multiple genes associated with cell-wall rigidity, supports the notion that cell-wall loosening facilitates primordium outgrowth [38,39]. This pattern is consistent with auxin-driven cell expansion.

A key contribution of this study is the spatiotemporal linkage between cell-wall remodeling and defense priming. Sustained upregulation of *PR-1* at several critical stages (days 0, 20, and 30), along with coordinated expression of defense-related regulators such as *WRKY* transcription factors and *RPM1*, suggests that ABT may transcriptionally prime plant defense pathways against potential pathogen challenge [40,41]. This coupling of structural remodeling with defense-associated transcriptional activation may partly explain the improved performance of ABT-treated cuttings.

## 5. Conclusions

This study advances our understanding of how composite growth regulators function during rooting. Our data suggest that ABT is associated with shifts in energy-related regulation during adventitious root formation, potentially mediated by coordinated jasmonate signal attenuation (via *CYP94C1*) and defense priming (e.g., *PR-1/WRKY*). These results provide transcriptomic evidence relevant to the context-dependent role of jasmonate in difficult-to-root species. In addition, the oscillatory expression of *ERF1/2* implies stage-specific ethylene regulation and may contribute to the transition between developmental and defense programs, thereby extending current concepts of temporal ethylene control during AR formation.

Based on these findings, we propose a three-tier regulatory framework—“hormone homeostasis–cell-wall remodeling–defense priming”—to explain the coordination between root induction and defense-associated responses during clonal propagation of forest trees. Nevertheless, this study is limited by its focus on transcriptomic dynamics in cortical tissues; future work should incorporate spatially resolved validation (e.g., in situ approaches or spatial transcriptomics) to localize cell-type-specific expression. Moreover, the interaction between the IAA and NAA components within ABT remains to be clarified, potentially through genetic analyses using hormone-related mutants. Despite these limitations, the key modules (e.g., ME3/ME8/ME9) and candidate genes (*EXPA11*, *BAT1*, and *RBOHB*) identified here provide promising molecular targets for mulberry improvement, and the proposed model may inform efforts to enhance vegetative propagation in other forest tree species.

## Data Availability

The raw sequencing data presented in this study are available in the NCBI Sequence Read Archive (SRA) at https://www.ncbi.nlm.nih.gov/sra under BioProject accession PRJNA1417371 (accession date: 1 February 2026).

## Supplementary Materials

The following supporting information can be downloaded at https://www.mdpi.com/article/10.3390/cimb48030299/s1.
