# Supplementary material for: ABT Promotes Adventitious Root Formation in Mulberry Cuttings by Coordinating Hormonal Homeostasis and Defense Priming
Source: Curr Issues Mol Biol. 2026 Mar 11;48(3):299. doi: 10.3390/cimb48030299 (PMC13025735; doi:10.3390/cimb48030299)
Supplement: Supplementary file 1 [file cimb-48-00299-s001.zip › R code.pdf]

Differentially expressed genes were identified using the DESeq2 package in R:

```
pkgs <- c("DESeq2", "tidyverse")
to_install <- pkgs[!pkgs %in% rownames(installed.packages())]
if (length(to_install) > 0) install.packages(to_install)

library(DESeq2)
library(tidyverse)
# ---- 1) Read counts matrix ----
count_file <- "counts_matrix.csv"    # <- change
cts <- read.csv(count_file, header = TRUE, check.names = FALSE)
# If first column is gene ID:
rownames(cts) <- cts[[1]]
cts <- cts[, -1, drop = FALSE]
# Ensure integer matrix
cts <- as.matrix(cts)
mode(cts) <- "numeric"
cts <- round(cts)    # DESeq2 expects counts; if already integer, fine.
# ---- 2) Sample metadata ----
# sample_id must match colnames(cts)
coldata <- tibble(sample_id = colnames(cts)) %>%
  mutate(
    treatment = str_extract(sample_id, "^(CK|ABT)"),
    time      = str_extract(sample_id, "(0d|10d|20d|30d)"),
    replicate = str_extract(sample_id, "R\\d+$")
  )
coldata <- coldata %>%
  mutate(
    treatment = factor(treatment, levels = c("CK", "ABT")),
    time      = factor(time, levels = c("0d","10d","20d","30d"))
  ) %>%
  column_to_rownames("sample_id")
# ---- 3) Basic gene filtering (optional but recommended) ----
# Keep genes with at least 10 counts in >= 3 samples
keep <- rowSums(cts >= 10) >= 3
cts_f <- cts[keep, ]
# ---- 4) Function: run DESeq2 within each time point ----
run_deseq_timepoint <- function(time_point) {
  sel <- rownames(coldata)[coldata$time == time_point]
  dds <- DESeqDataSetFromMatrix(
    countData = cts_f[, sel, drop = FALSE],
    colData   = coldata[sel, , drop = FALSE],
    design    = ~ treatment
  )
  dds <- DESeq(dds)
```

```
  res <- results(dds, contrast = c("treatment", "ABT", "CK")) %>%
    as.data.frame() %>%
    rownames_to_column("gene_id") %>%
    mutate(time = time_point)
  # Apply your thresholds: |log2FC| >= 1 and P <= 0.05
  deg <- res %>%
    filter(!is.na(pvalue)) %>%
    filter(abs(log2FoldChange) >= 1, pvalue <= 0.05) %>%
    arrange(pvalue)
  list(res_all = res, deg = deg)
}
time_points <- c("0d","10d","20d","30d")
out <- lapply(time_points, run_deseq_timepoint)
names(out) <- time_points
# ---- 5) Save results ----
dir.create("DEG_DESeq2_by_time", showWarnings = FALSE)
for (tp in time_points) {
  write.csv(out[[tp]]$res_all,
            file = file.path("DEG_DESeq2_by_time", paste0("DESeq2_all_", tp,
"_ABT_vs_CK.csv")),
            row.names = FALSE)
  write.csv(out[[tp]]$deg,
            file = file.path("DEG_DESeq2_by_time", paste0("DESeq2_DEG_", tp,
"_ABT_vs_CK_log2FC1_p0.05.csv")),
            row.names = FALSE)
  cat(tp, "DEGs:", nrow(out[[tp]]$deg), "\n")
}
# ---- 6) Combine all DEGs into one table (optional) ----
deg_all <- bind_rows(lapply(out, `[[`, "deg"))
write.csv(deg_all, file.path("DEG_DESeq2_by_time", "DEG_all_timepoints_combined.csv"),
row.names = FALSE)
```

PCA analysis:

```
pkgs <- c("tidyverse", "ggrepel")
to_install <- pkgs[!pkgs %in% rownames(installed.packages())]
if (length(to_install) > 0) install.packages(to_install)
library(tidyverse)
library(ggrepel)
# Expectation: rows = genes, columns = samples
# First column = gene_id (optional). If your file already has rownames, set row.names=1.
expr_file <- "expression_matrix.csv"    # <- change to your file
expr <- read.csv(expr_file, header = TRUE, check.names = FALSE)
# If the first column is gene id, set it as rownames
# (If your file already has rownames, comment these two lines)
```

```r
rownames(expr) <- expr[[1]]
expr <- expr[, -1, drop = FALSE]
# Ensure numeric
expr[] <- lapply(expr, function(x) as.numeric(as.character(x)))
expr <- as.matrix(expr)
# IMPORTANT: sample_id must match colnames(expr)
# Fill in your exact 24 sample names
sample_info <- tibble(
  sample_id = colnames(expr),
  # Example naming scheme: CK_0d_R1, ABT_0d_R1, ...
  # If you already encode info in sample names, see the parsing code below.
  treatment = NA_character_,
  time      = NA_character_,
  replicate = NA_character_
)

# ---- Option A (recommended): parse from sample names
# Assumes sample names like: CK_0d_R1, ABT_10d_R2, etc.
# Adjust the regex if your naming differs.
sample_info <- sample_info %>%
  mutate(
    treatment = str_extract(sample_id, "^(CK|ABT)"),
    time      = str_extract(sample_id, "(0d|10d|20d|30d)"),
    replicate = str_extract(sample_id, "R\\d+$")
  )
# If parsing fails (NAs), you can manually set columns:
# sample_info$treatment <- c(rep("CK", 12), rep("ABT", 12))   # example
# sample_info$time <- rep(c("0d","10d","20d","30d"), each=6) # example
# sample_info$replicate <- rep(paste0("R",1:3), times=8)      # example
# Make factors with desired order
sample_info <- sample_info %>%
  mutate(
    treatment = factor(treatment, levels = c("CK", "ABT")),
    time      = factor(time, levels = c("0d", "10d", "20d", "30d"))
  )
# 3) Preprocessing for PCA
# Common choices:
# - If data are raw counts: use log2(count+1) or variance-stabilizing transform (DESeq2).
# - If data are TPM/FPKM: log2(TPM/FPKM + 1) is typical.
# - Then filter low-expression genes and optionally scale.
# Here: log2(x + 1)
expr_log <- log2(expr + 1)
# Filter genes with low expression across samples (tune thresholds as needed)
# Keep genes expressed (log2 > 1) in at least 6 samples, for example
```

```
keep <- rowSums(expr_log > 1) >= 6
expr_filt <- expr_log[keep, , drop = FALSE]
# PCA is done on samples, so transpose: samples x genes
# If your matrix is already samples x genes, REMOVE t() below.
mat_for_pca <- t(expr_filt)
# Scale features (genes) so PCA isn't dominated by high-variance genes
# This is standard when using expression matrices.
pca <- prcomp(mat_for_pca, center = TRUE, scale. = TRUE)
# Variance explained
var_explained <- (pca$sdev^2) / sum(pca$sdev^2)
pc1 <- round(var_explained[1] * 100, 2)
pc2 <- round(var_explained[2] * 100, 2)
# 4) Prepare PCA dataframe
pca_df <- as.data.frame(pca$x[, 1:5, drop = FALSE]) %>%
  rownames_to_column("sample_id") %>%
  left_join(sample_info, by = "sample_id")
# 5) Plot PCA
# Color by treatment, shape by time
p <- ggplot(pca_df, aes(x = PC1, y = PC2, color = treatment, shape = time)) +
  geom_point(size = 3, alpha = 0.9) +
  ggrepel::geom_text_repel(aes(label = sample_id), size = 3, max.overlaps = 50) +
  labs(
    title = "PCA of 24 samples (expression matrix)",
    x = paste0("PC1 (", pc1, "%)"),
    y = paste0("PC2 (", pc2, "%)")
  ) +
  theme_bw(base_size = 12) +
  theme(
    plot.title = element_text(hjust = 0.5),
    legend.position = "right"
  )
print(p)
# Optional: facet by time (often nice to see separation within each time)
p_facet <- ggplot(pca_df, aes(x = PC1, y = PC2, color = treatment)) +
  geom_point(size = 3, alpha = 0.9) +
  facet_wrap(~ time) +
  labs(
    title = "PCA (faceted by time)",
    x = paste0("PC1 (", pc1, "%)"),
    y = paste0("PC2 (", pc2, "%)")
  ) +
  theme_bw(base_size = 12) +
  theme(plot.title = element_text(hjust = 0.5))
print(p_facet)
```

```
# 6) Save outputs
write.csv(pca_df, "PCA_scores_with_metadata.csv", row.names = FALSE)
ggsave("PCA_PC1_PC2.png", p, width = 9, height = 6, dpi = 300)
ggsave("PCA_PC1_PC2_facet_time.png", p_facet, width = 10, height = 6, dpi = 300)
```

Weighted gene coexpression network analysis:

```
pkgs <- c("WGCNA", "data.table", "tidyverse")
to_install <- pkgs[!pkgs %in% rownames(installed.packages())]
if (length(to_install) > 0) install.packages(to_install)
library(WGCNA)
library(data.table)
library(tidyverse)
# Speed up (optional)
allowWGCNAThreads()
expr_file <- "expression_matrix.csv"    # <- change
expr <- read.csv(expr_file, header = TRUE, check.names = FALSE)
# If first column is gene id, set rownames and drop it
rownames(expr) <- expr[[1]]
expr <- expr[, -1, drop = FALSE]
# Convert to numeric matrix
expr[] <- lapply(expr, function(x) as.numeric(as.character(x)))
expr <- as.matrix(expr)
# If your matrix is samples x genes, uncomment next line:
# expr <- t(expr)
# ---- 2) Log transform (adjust based on your data type) ----
# For counts/FPKM/TPM, log2(x+1) is common before WGCNA
datExpr0 <- t(log2(expr + 1))    # WGCNA wants samples x genes
# ---- 3) Basic QC: remove bad samples/genes ----
gsg <- goodSamplesGenes(datExpr0, verbose = 3)
if (!gsg$allOK) {
  if (sum(!gsg$goodGenes) > 0) cat("Removing genes:", paste(colnames(datExpr0)[!gsg$goodGenes], collapse=", "), "\n")
  if (sum(!gsg$goodSamples) > 0) cat("Removing samples:", paste(rownames(datExpr0)[!gsg$goodSamples], collapse=", "), "\n")
  datExpr0 <- datExpr0[gsg$goodSamples, gsg$goodGenes]
}
# Optional: sample clustering to detect outliers
sampleTree <- hclust(dist(datExpr0), method = "average")
pdf("WGCNA_01_sampleClustering.pdf", width = 10, height = 6)
plot(sampleTree, main = "Sample clustering", xlab = "", sub = "", cex.lab = 1.2)
dev.off()

# ---- 4) Gene filtering (recommended for RNA-seq to reduce noise) ----
# Filter low-variance genes (keep top X% variance) or low-expression
geneVar <- apply(datExpr0, 2, var, na.rm = TRUE)
```

```
# Keep top 25% most variable genes (tune as needed: 0.25/0.5 etc.)
keepFrac <- 0.25
keepN <- max(50, floor(length(geneVar) * keepFrac))    # keep at least 50 genes
keepGenes <- names(sort(geneVar, decreasing = TRUE))[1:keepN]
datExpr <- datExpr0[, keepGenes]

cat("Genes kept for WGCNA:", ncol(datExpr), "\n")
cat("Samples:", nrow(datExpr), "\n")
samples <- rownames(datExpr)
traits <- data.frame(sample_id = samples)

traits$treatment <- stringr::str_extract(traits$sample_id, "^(CK|ABT)")
traits$time <- stringr::str_extract(traits$sample_id, "(0d|10d|20d|30d)")
traits$replicate <- stringr::str_extract(traits$sample_id, "R\\d+$")
traits$treatment_num <- ifelse(traits$treatment == "ABT", 1, 0)

time_levels <- c("0d","10d","20d","30d")
traits$time <- factor(traits$time, levels = time_levels, ordered = TRUE)
traits$time_num <- as.numeric(traits$time)    # 1..4 (monotonic trend)

# Also create one-hot encoding for time (optional, often useful)
time_dummies <- model.matrix(~ time - 1, data = traits) %>% as.data.frame()
colnames(time_dummies) <- gsub("^time", "T", colnames(time_dummies))    # e.g., T0d T10d ...
traitData <- cbind(traits[, c("treatment_num","time_num")], time_dummies)
rownames(traitData) <- traits$sample_id

# Reorder to match datExpr
traitData <- traitData[rownames(datExpr), , drop = FALSE]

# ---- 6) Choose soft-thresholding power (beta) ----
powers <- c(seq(1, 10, 1), seq(12, 30, 2))
sft <- pickSoftThreshold(datExpr, powerVector = powers, networkType = "signed", verbose = 5)

pdf("WGCNA_02_softThreshold.pdf", width = 10, height = 5)
par(mfrow = c(1,2))
plot(sft$fitIndices[,1], -sign(sft$fitIndices[,3]) * sft$fitIndices[,2],
     xlab = "Soft Threshold (power)", ylab = "Scale Free Topology Model Fit, signed R^2",
     type = "n", main = "Scale independence")
text(sft$fitIndices[,1], -sign(sft$fitIndices[,3]) * sft$fitIndices[,2],
     labels = powers, cex = 0.8, col = "red")
abline(h = 0.8, col = "blue", lty = 2)

plot(sft$fitIndices[,1], sft$fitIndices[,5],
     xlab = "Soft Threshold (power)", ylab = "Mean Connectivity",
```

```
        type = "n", main = "Mean connectivity")
text(sft$fitIndices[,1], sft$fitIndices[,5], labels = powers, cex = 0.8, col = "red")
dev.off()

# Pick beta: lowest power achieving R^2 >= 0.8 if possible
fit <- -sign(sft$fitIndices[,3]) * sft$fitIndices[,2]
candidate <- sft$fitIndices[fit >= 0.8, 1]
softPower <- if (length(candidate) > 0) min(candidate) else 12    # fallback
cat("Chosen softPower:", softPower, "\n")

# ---- 7) Build network and detect modules ----
# Use blockwiseModules (recommended)
net <- blockwiseModules(
  datExpr,
  power = softPower,
  networkType = "signed",
  TOMType = "signed",
  minModuleSize = 50,
  reassignThreshold = 0,
  mergeCutHeight = 0.2,          # merge threshold ~0.8 similarity (since 1-0.2=0.8)
  numericLabels = FALSE,
  pamRespectsDendro = TRUE,
  saveTOMs = TRUE,
  saveTOMFileBase = "WGCNA_TOM",
  verbose = 3
)

moduleColors <- net$colors
MEs <- net$MEs
geneTree <- net$dendrograms[[1]]

pdf("WGCNA_03_geneDendrogram_modules.pdf", width = 12, height = 6)
plotDendroAndColors(
  geneTree,
  moduleColors[net$blockGenes[[1]]],
  "Module colors",
  dendroLabels = FALSE,
  hang = 0.03,
  addGuide = TRUE,
  guideHang = 0.05
)
dev.off()

# ---- 8) Module-trait relationships ----
```

```
# Ensure MEs rownames match samples
MEs0 <- orderMEs(MEs)
moduleTraitCor <- cor(MEs0, traitData, use = "p")
moduleTraitPvalue <- corPvalueStudent(moduleTraitCor, nSamples = nrow(datExpr))

# Heatmap
textMatrix <- paste0(signif(moduleTraitCor, 2), "\n(",
                     signif(moduleTraitPvalue, 1), ")")
dim(textMatrix) <- dim(moduleTraitCor)

pdf("WGCNA_04_moduleTrait_heatmap.pdf", width = 12, height = 8)
par(mar = c(6, 8.5, 3, 3))
labeledHeatmap(
  Matrix = moduleTraitCor,
  xLabels = colnames(traitData),
  yLabels = names(MEs0),
  ySymbols = names(MEs0),
  colorLabels = FALSE,
  colors = blueWhiteRed(50),
  textMatrix = textMatrix,
  setStdMargins = FALSE,
  cex.text = 0.7,
  zlim = c(-1,1),
  main = "Module-trait relationships\ncor (p-value)"
)
dev.off()

# Save correlations
write.csv(moduleTraitCor, "WGCNA_moduleTraitCor.csv")
write.csv(moduleTraitPvalue, "WGCNA_moduleTraitPvalue.csv")

# ---- 9) Export module gene lists ----
geneInfo <- data.frame(
  gene_id = colnames(datExpr),
  module = moduleColors,
  stringsAsFactors = FALSE
)
write.csv(geneInfo, "WGCNA_geneModule_assignment.csv", row.names = FALSE)

# ---- 10) Hub gene candidates for a module of interest ----
# Choose a module (e.g., "turquoise") and compute:
# - Module Membership (kME): cor(gene expression, module eigengene)
# - Gene Significance (GS): cor(gene expression, trait)
module_of_interest <- "turquoise"    # <- change to your module name (color)
```

```
trait_of_interest <- "treatment_num" # <- change to one column in traitData

if (module_of_interest %in% unique(moduleColors)) {
  modGenes <- (moduleColors == module_of_interest)
  ME_col <- MEs0[, paste0("ME", module_of_interest)]

  kME <- cor(datExpr[, modGenes], ME_col, use = "p")
  GS   <- cor(datExpr[, modGenes], traitData[[trait_of_interest]], use = "p")

  hub <- data.frame(
    gene_id = colnames(datExpr)[modGenes],
    module = module_of_interest,
    kME = as.numeric(kME),
    GS = as.numeric(GS)
  ) %>%
    arrange(desc(abs(kME)), desc(abs(GS)))

  write.csv(hub, paste0("WGCNA_hubCandidates_", module_of_interest, "_", trait_of_interest,
".csv"),
            row.names = FALSE)
} else {
  cat("Module", module_of_interest, "not found. Available modules:\n")
  print(unique(moduleColors))
}

# ---- 11) Save eigengenes ----
ME_out <- data.frame(sample_id = rownames(MEs0), MEs0, check.names = FALSE)
write.csv(ME_out, "WGCNA_moduleEigengenes.csv", row.names = FALSE)

cat("WGCNA finished.\nOutputs:\n",
    "- PDFs: sample clustering, soft-threshold, dendrogram, module-trait heatmap\n",
    "- CSVs: module-trait cor/p, gene-module assignment, module eigengenes, hub
candidates\n")
```

KEGG Orthology enrichment

```
pkgs <- c("clusterProfiler", "tidyverse", "readr")
to_install <- pkgs[!pkgs %in% rownames(installed.packages())]
if (length(to_install) > 0) install.packages(to_install)

library(clusterProfiler)
library(tidyverse)
library(readr)
```

```
# ---- 1) Load gene->KO mapping ----
gene2ko <- read_csv("annotation_ko.csv")    # <- change
# Expect columns: gene_id, KO (e.g., K01915)
gene2ko <- gene2ko %>%
  filter(!is.na(KO)) %>%
  mutate(KO = str_extract(KO, "K\\d{5}")) %>%
  filter(!is.na(KO)) %>%
  distinct(gene_id, KO)

# ---- 2) Helper: run enrichKEGG with organism = "ko" ----
run_kegg_ko <- function(deg_file, out_prefix) {
  deg <- read_csv(deg_file)

  ko_list <- deg %>%
    inner_join(gene2ko, by = "gene_id") %>%
    pull(KO) %>%
    unique()

  if (length(ko_list) < 10) {
    message("Too few KO-mapped genes for ", out_prefix, ": ", length(ko_list))
    return(NULL)
  }

  ek <- enrichKEGG(
    gene          = ko_list,
    organism      = "ko",          # KO universe
    keyType       = "kegg",        # K numbers
    pvalueCutoff  = 0.05,
    pAdjustMethod = "BH",
    qvalueCutoff  = 1
  )

  if (is.null(ek) || nrow(as.data.frame(ek)) == 0) {
    message("No enriched KEGG terms for ", out_prefix)
    return(NULL)
  }

  # Save
  write.csv(as.data.frame(ek), paste0(out_prefix, "_KEGG_KO_enrichment.csv"), row.names =
FALSE)

  # Plots
  pdf(paste0(out_prefix, "_KEGG_KO_dotplot.pdf"), width = 9, height = 6)
```

```
  print(dotplot(ek, showCategory = 20) + ggtitle(out_prefix))
  dev.off()

  pdf(paste0(out_prefix, "_KEGG_KO_barplot.pdf"), width = 9, height = 6)
  print(barplot(ek, showCategory = 20) + ggtitle(out_prefix))
  dev.off()

  return(ek)
}

# ---- 3) Run per time point ----
dir.create("KEGG_KO", showWarnings = FALSE)
setwd("KEGG_KO")

ek0   <- run_kegg_ko("../DEG_0d.csv",   "0d_ABT_vs_CK")
ek10 <- run_kegg_ko("../DEG_10d.csv", "10d_ABT_vs_CK")
ek20 <- run_kegg_ko("../DEG_20d.csv", "20d_ABT_vs_CK")
ek30 <- run_kegg_ko("../DEG_30d.csv", "30d_ABT_vs_CK")
```

GO Orthology enrichment

```
pkgs <- c("clusterProfiler", "tidyverse", "readr", "stringr")
to_install <- pkgs[!pkgs %in% rownames(installed.packages())]
if (length(to_install) > 0) install.packages(to_install)

library(clusterProfiler)
library(tidyverse)
library(readr)
library(stringr)

# ------------------------------
# 1) Load gene -> GO mapping
# ------------------------------
# annotation_go.csv must contain: gene_id, GO
# GO can be single (GO:xxxxxxx) or multiple separated by ; , | space
gene2go_raw <- read_csv("annotation_go.csv")   # <- change

# Normalize to TERM2GENE (GO -> gene) format required by enricher()
term2gene <- gene2go_raw %>%
  transmute(
    gene_id = as.character(gene_id),
    GO = as.character(GO)
  ) %>%
  filter(!is.na(gene_id), !is.na(GO)) %>%
  mutate(GO = str_replace_all(GO, "\\s+", "")) %>%
```

```
  separate_rows(GO, sep = "[;|, ]+") %>%                # split multiple GO terms
  filter(str_detect(GO, "^GO:\\d{7}$")) %>%          # keep valid GO IDs
  distinct(GO, gene_id) %>%
  rename(term = GO, gene = gene_id)

# Optional: create TERM2NAME (GO -> name) if you have names in annotation
# If you don't have GO term names, clusterProfiler will still run,
# but plots will show GO IDs as Description.
# If your annotation_go.csv has a "GO_name" column, uncomment below:
# term2name <- gene2go_raw %>%
#     transmute(term = GO, name = GO_name) %>%
#     distinct(term, name)

# ------------------------------
# 2) Define background universe
# ------------------------------
# Recommended: all genes that passed filtering / were tested in DEG analysis
# If you have a "tested_genes.txt" (one gene_id per line), load it here:
# universe_genes <- read_lines("tested_genes.txt")

# If not, use all genes present in the annotation as background:
universe_genes <- unique(term2gene$gene)

# ------------------------------
# 3) Helper function: run GO enrichment for one DEG file
# ------------------------------
run_go_enrich <- function(deg_file, out_prefix, term2gene, universe_genes,
                          p_cut = 0.05, q_cut = 1, minGSSize = 10, maxGSSize = 500) {
  deg <- read_csv(deg_file)

  # Ensure gene_id column exists
  if (!("gene_id" %in% colnames(deg))) {
    stop("DEG file must contain a column named 'gene_id': ", deg_file)
  }

  gene_list <- deg %>%
    transmute(gene_id = as.character(gene_id)) %>%
    filter(!is.na(gene_id)) %>%
    pull(gene_id) %>%
    unique()

  # Intersect with universe
  gene_list <- intersect(gene_list, universe_genes)
```

```
  if (length(gene_list) < 10) {
    message("Too few genes for GO enrichment in ", out_prefix, ": ", length(gene_list))
    return(NULL)
  }

  ego <- enricher(
    gene          = gene_list,
    universe      = universe_genes,
    TERM2GENE     = term2gene,
    pvalueCutoff  = p_cut,
    pAdjustMethod = "BH",
    qvalueCutoff  = q_cut,
    minGSSize     = minGSSize,
    maxGSSize     = maxGSSize
  )

  if (is.null(ego) || nrow(as.data.frame(ego)) == 0) {
    message("No enriched GO terms for ", out_prefix)
    return(NULL)
  }

  # Save table
  write.csv(as.data.frame(ego), paste0(out_prefix, "_GO_enrichment.csv"), row.names = FALSE)

  # Plots
  pdf(paste0(out_prefix, "_GO_dotplot.pdf"), width = 10, height = 6)
  print(dotplot(ego, showCategory = 20) + ggtitle(out_prefix))
  dev.off()

  pdf(paste0(out_prefix, "_GO_barplot.pdf"), width = 10, height = 6)
  print(barplot(ego, showCategory = 20) + ggtitle(out_prefix))
  dev.off()

  return(ego)
}

# -----------------------------
# 4) Run enrichment per time point
# -----------------------------
dir.create("GO_enrichment", showWarnings = FALSE)
setwd("GO_enrichment")

ego0  <- run_go_enrich("../DEG_0d.csv",  "0d_ABT_vs_CK",  term2gene, universe_genes)
ego10 <- run_go_enrich("../DEG_10d.csv", "10d_ABT_vs_CK", term2gene, universe_genes)
```

```
ego20 <- run_go_enrich("../DEG_20d.csv", "20d_ABT_vs_CK", term2gene, universe_genes)
ego30 <- run_go_enrich("../DEG_30d.csv", "30d_ABT_vs_CK", term2gene, universe_genes)

cat("GO enrichment finished. Results saved to GO_enrichment/ \n")
```
