# Supplementary material for: ABT Promotes Adventitious Root Formation in Mulberry Cuttings by Coordinating Hormonal Homeostasis and Defense Priming
Source: Curr Issues Mol Biol. 2026 Mar 11;48(3):299. doi: 10.3390/cimb48030299 (PMC13025735; doi:10.3390/cimb48030299)
Supplement: Supplementary file 1 [file cimb-48-00299-s001.zip › supplementary materials.pdf]

Fig. S1

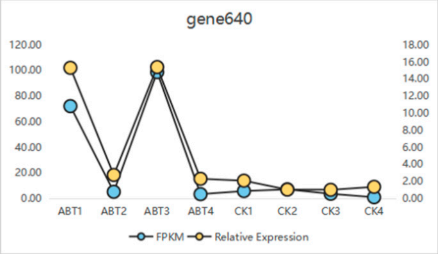

（a）

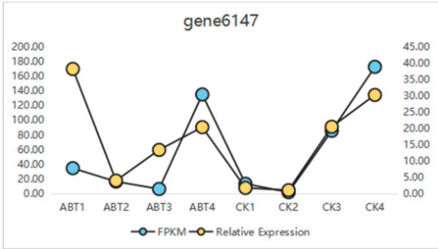

（b）

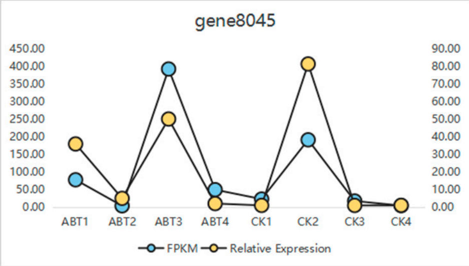

（c）

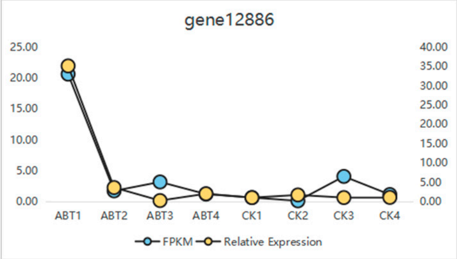

（d）

Fig. S1:(a) gene640 FPKM and relative expression (b) gene6147 FPKM and relative expression (c) gene8045 FPKM and relative expression (d) gene12886 FPKM and relative expressionFour genes were randomly selected for RT-qPCR analysis. (Expression levels were normalized to the Actin gene as an internal reference, and relative expression levels were calculated using the $2^{-}\Delta\Delta Ct$ method. The results showed good consistency with the transcriptomic data)
